# Global Health, Geographical Contingency, and Contingent Geographies

**DOI:** 10.1080/24694452.2016.1140017

**Published:** 2016-04-06

**Authors:** Clare Herrick

**Affiliations:** ^a^ Department of Geography, King's College London

**Keywords:** alcohol, Botswana, complexity, contingency, geography, global health, alcohol, Botsuana, complejidad, contingencia, geografía, salud global

## Abstract

Health geography has emerged from under the “shadow of the medical” to become one of the most vibrant of all the subdisciplines. Yet, this success has also meant that health research has become increasingly siloed within this subdisciplinary domain. As this article explores, this represents a potential lost opportunity with regard to the study of global health, which has instead come to be dominated by anthropology and political science. Chief among the former's concerns are exploring the gap between the programmatic intentions of global health and the unintended or unanticipated consequences of their deployment. This article asserts that recent work on contingency within geography offers significant conceptual potential for examining this gap. It therefore uses the example of alcohol taxation in Botswana, an emergent global health target and tool, to explore how *geographical* contingency and the emergent, contingent geographies that result might help counter the prevailing tendency for geography to be side-stepped within critical studies of global health. At the very least, then, this intervention aims to encourage reflection by geographers on how to make explicit the all-too-often implicit links between their research and global health debates located outside the discipline.

## Abstract (zh)

健康地理学, 已脱离了 “医学阴影”, 浮现成为最活跃的次领域之一。但此般成功, 却也同时意味着健康研究逐渐在此一次领域范畴中受到孤立。如同本文所探讨的一般, 这代表着研究全球健康的潜在契机之丧失, 并成为人类学与政治科学所主宰的领域。前者的考量中, 最主要在于探讨全球健康的计画目的, 与其施展后的不经意或非预期结果之间的落差。本文主张, 晚近地理学中有关偶然性的研究, 提供了检视此一落差的重要概念潜能。本文因而使用博茨瓦纳所施行的酒税案例——一个全球健康的目标与工具——来探讨地理偶然性及其导致的浮现中且偶发的地理, 如何可能协助对抗全球健康的批判研究避开地理学的盛行趋势。此般介入的目的, 至少在于鼓励地理学者反思如何使其研究与领域之外的全球健康辩论之间过于隐晦的连结变得清晰可见。

## Abstract (fr)

La geografía de la salud ha emergido desde la “sombra de la geografía médica” para convertirse en una de las subdisciplinas más dinámicas. Sin embargo, este éxito ha significado también que la investigación sobre la salud crecientemente se haya enclaustrado dentro de este dominio subdisciplinario. Como lo explora este artículo, esto representa una potencial pérdida de oportunidad en lo que concierne al estudio de la salud global, que alternativamente ha llegado a caer dentro del dominio de la antropología y la ciencia política. Importante entre las preocupaciones de la primera son la exploración de la brecha entre las intenciones programáticas de la salud global y las consecuencias no intencionales o imprevistas de su despliegue. Este artículo sostiene que el trabajo reciente sobre contingencia dentro de la geografía presenta un significativo potencial conceptual para el examen de esa brecha. Este utiliza por lo eso el ejemplo de la tributación del alcohol en Botsuana, un propósito global emergente de la salud y herramienta para explorar de qué manera la contingencia geográfica y las geografías emergentes y contingentes que resultan podrían ayudar a contrarrestar la tendencia prevaleciente en geografía de ser soslayada dentro de los estudios críticos de la salud global. Como mínimo, entonces, esta intervención apunta a estimular la reflexión de los geógrafos sobre cómo hacer explícitos los más que frecuentes enlaces implícitos entre su investigación y los debates globales sobre salud ubicados fuera de la disciplina.

This article emerges from two concerns: first, that the potential health significance of much geographical research is being footnoted rather than foregrounded; second, that this trend is particularly acute in relation to the contemporary field of critical global health studies. The first concern originates, somewhat ironically, in the hugely successful efforts of health geographers in carving out a distinct subdisciplinary identity (Andrews et al. 2012). Yet, the price of this success is that research on health is too often siloed within this relatively small subdisciplinary realm.[Fn en0001] This means that the potential of “health” as a powerfully holistic conceptual and empirical vehicle by which to interrogate a plethora of social, economic, political, and biophysical processes, trends, and states remains underrealized (Petersen and Lupton 1996). The second concern originates in the observation that despite “emerging work in health geography … which calls for a rethinking of the meaning of global health” (Dunn, Le Mare, and Makungu 2015, 17), such advances have been eclipsed by anthropological and political science engagements with this novel field (see, e.g., Pfeiffer and Nichter 2008; Elbe 2010). Indeed, geography has been sidelined in a way that ignores how the compulsion to do and understand global health emerges largely from contexts that the discipline actively pioneers: urbanization, globalization, political ecology, security, risk, vulnerability, resilience, geopolitics, more-than-human natures, foodscapes, culture, governance, development, and the environment, to name but a few (Herrick 2014). Furthermore, global health brings with it an innate, powerful, and politicizing spatial logic that forms the core of the geographical armory but is now being touted as theoretical novelty by other social sciences (Janes and Corbett 2009).

My intervention comes at a time of growing interest in how to make better theory to explore and explain the “obscure object” of global health (Fassin 2012). Indeed, celebrated physician and anthropologist Paul Farmer and colleagues made a clear argument for the application of social theory to help global health's transformation from an often ephemeral assemblage of knowledge, actors, practices, and politics into a coherent “discipline” (Farmer et al. 2013). Such theories are needed, they argued, to better understand the production, legitimation, and experiences of global health, as well as the processes by which these are made meaningful in a variety of social, spatial, economic, and political contexts. Theories are also needed to account for global health's failures, as much as to explain its successes. Geographical perspectives could then help respond to mounting calls for greater constructive (rather than critical for critique's sake) social scientific attention not only to the policies and praxis of global health but also to the disjunctures between intentions and effects (Kleinman 2010; Holmes, Greene, and Stonington 2014). Taking these disjunctures as a starting point, I argue here that contingency—an often used but underelaborated phrase in anthropological engagements with global health (Panter-Brick, Eggerman, and Tomlinson 2014)—and, more crucially, geographical contingency, offers a conceptual opportunity to better elide geographical research with transdisciplinary critical studies of global health. This elision offers great potential, for as Barry (2014) noted, “one of the virtues of Geography is that contingency is at the heart of its concerns” (1).

To further develop this contribution, I proceed in three parts. First, by way of context, I explore what global health is and claims to be. I then turn to examine the domination of the critical global health field by anthropology as a way of explaining not only the relative sidelining of geography but also why so many potential geographical contributions to (global) health debates have failed to be positioned as such within and outside the discipline. I then turn to the concept of contingency to explore how this might help shed light on the common anthropological concern with why the imposition of health interventions so often produces unintended and unanticipated consequences. To add empirical depth to this exploration of contingency, I work through the example of alcohol taxation, an emergent realm of global health activity that cuts across manifold spatial scales, legal jurisdictions, trade agreements, epidemiological knowledge, public health advocacy, and cultural significance. Drawing on recent research on the consequences of Botswana's divisive alcohol levy, I critically reflect on the significance of *geographical contingency*—or the sociospatial relations and conditions inherent within the unfolding of contingency—to the genesis of multiple, variegated, and unanticipated effects of alcohol control measures. I then argue that these effects can be understood as a series of *contingent geographies*—or sociospatial formations and processes that emerge in dynamic and unpredictable ways—before examining the implications of these contingencies for a newly theorized, interdisciplinary global health.

## What Is Global Health?

Defining any unified field of global health has long proved to be a significant challenge (Koplan et al. 2009), yet only recently has this been deemed a problem. Indeed, as Fassin (2012) suggested, “until now, the expression global health seems to have been self-evident, as if we should all know what it signifies and share what it refers to” (100). The field is now subject to critique for its lack of both a coherent theoretical underpinning and status as a discipline, however. Although this argument cannot be dissociated from universities' current obsession with global health programs (Macfarlane, Jacobs, and Kaaya 2008; Crane 2011), it is still the case that people generally engage with global health only indirectly through its rationales, problem frames, justifications, programs, and evidence base. It follows then that “despite the appearance of a shared moral and technical project, global health is not a unified field … it is not clear precisely what the term means” (Lakoff 2010, 59). In brief, this “comparatively new multilateral enterprise” (Garrett 2013, 2), has been largely guided by the World Health Organization (WHO) and more recently the World Bank and a plethora of philanthropic enterprises (McGoey 2015). It involves the transfer of knowledge and resources from Global North to Global South, a variety of efforts to act on and reduce the global burden of disease, and a particular concern for and financial investment in the infectious disease triumvirate of HIV/AIDS, malaria, and tuberculosis (Ingram 2009; Koplan et al. 2009).

Global health is further characterized by its multilayered and complex architecture (Ruger 2007). By this I mean the assemblage of biomedical knowledge, technologies, agendas, initiatives, objectives, data, and politics articulated through the interconnections among nation-states, international organizations, private companies, nongovernmental organizations (NGOs), regional trade agreements, localities, philanthropies, lay people, and experts and through an array of projects, interventions, and surveillance enterprises. At the last count, this involved “26 UN agencies, 20 global and regional funds, 40 bilateral donors, and 90 global health initiatives” (Panter-Brick, Eggerman, and Tomlinson 2014, 2). Global health is thus a dynamic and opportunistic enterprise with geopolitical context, funding streams, calculations of epidemiological need, and modes of deployment that are constantly shifting. This enterprise is, nevertheless, underpinned by a number of compulsions that coalesce (at least discursively) around the will to secure health for all in the most efficient way possible, redress fundamental inequities, deliver value for money, and tackle the mounting economic burden and security threats posed by disease and suffering (Koplan et al. 2009; Lakoff 2010).

At present, the global health landscape is overwhelmingly dominated by a metricized logic of technological “fixes,” efficiency, evidence, measurement, and evaluation (Adams 2013), led by (but not limited to) the Bill and Melinda Gates Foundation (BMGF). This Gates approach can often be grossly at odds with social scientific concern with the gap between programmatic intention, situated experience, and social, political, and economic contexts in many global health interventions (Adams, Burke, and Whitmarsh 2014; Storeng and Mishra 2014). The need to explain, manage, and close this gap—if only for the sake of greater efficiency—has not gone unnoticed and, in late 2014, Gates announced a new set of more socially minded “Grand Challenges.” Described by *The Economist* (“A New Challenge” 2014) as being “fuzzy” for their lack of scientific “specificity,” this partial concession to many of the social science critiques levelled at the BMGF represents a fascinating turn (“What Has the Gates Foundation Done?” 2008; McCoy et al. 2009). It also shows that although the policies, practices, successes, and failures of global health touch the everyday lives of countless individuals (Biehl and Petryna 2014), great tensions still remain between competing disciplinary truth claims about the nature, genesis, and experience of suffering. Amid this disciplinary antagonism, geography has failed to develop the same kind of critical conceptual mass that has allowed anthropologists to stake a unique claim to the global health field.

## Anthropological Ascendency and Geographical Opportunity

To date, medical anthropologists have arguably come to occupy the most compelling and persuasive niche in critical global health studies (Nguyen and Peschard 2003; see also Somatosphere.net; Pfeiffer and Nichter 2008; Farmer et al. 2013). Farmer, one such “global apostle of health” (Fassin 2012, 114), argued that global health must be understood as a biosocial enterprise in which illness emanates from multiple ecological scales (Farmer 1996b; Farmer et al. 2006; see also Gandy 2005). This approach has found favor as it decenters individual behavior as a default explanatory category and medicine as the sole cure and demands a series of more nuanced, contextualized, and situated analytical tools. The allied anthropological focus on “local biology” (see Lock and Nguyen 2010) represents an important counterpoint to the framing of global health as a (macro) problem of globalization that has prevailed in political science (Rowson et al. 2012). It also represents a critique of the epidemiological tendency to gloss over the lived experiences of health and illness as “anecdotal distraction” (Jeavons 2014, 462). So armed, anthropological engagements with global health have proven particularly powerful in arguing that the roots of poor health are not just “historically deep and geographically broad” (Farmer et al. 2013, 2) but must also be filtered through the critical frameworks of ethnographic methods and social theory (Pigg 2013). The assertion that ethnography is both an unparalleled “empirical lantern” (Biehl and Petryna 2014, 376) and the singularly most appropriate method for global health analysis has culminated in calls for a new methodological and social movement based on the mantra of ethnographic “slow research” (Adams, Burke, and Whitmarsh 2014). Such celebratory narratives of ethnographic (and thus anthropological) exceptionalism have been further entrenched by the shift from traditional studies of single localities to new, multisited and multiscalar ethnographies (Rajak 2011; Crane 2013; Fassin 2013) that are particularly well suited to global health research.

Despite anthropology's impressive ascendance, critiques of global health still remain relatively constrained regardless of discipline. This could be because, “like evidence-based medicine, the ideal of global health has assumed a certain rhetorical universality. … Just as few would claim to practice ‘evidence-free’ medicine, it is quite difficult in this era for anyone to argue against global health” (Holmes, Greene, and Stonington 2014, 475). Biehl and Petryna (2014) thus pointed out that “initiatives are booming … [yet] critical analyses of the social, political, and economic processes associated with this expanding field—an ‘open source anarchy’ on the ground—are still few and far between” (376). As momentum gathers behind the post-2015 Sustainable Development Goals (SDGs), however, criticism of the current architecture, transparency, and funding priorities of global health is starting to mount. This is particularly true in relation to global health's relative silence on noncommunicable diseases (NCDs; Livingston 2012; Marrero, Bloom, and Adashi 2012), despite the Global Burden of Disease Study showing that they now cause two thirds of global mortality (Reubi, Herrick, and Brown 2015). Moreover, and crucially for the sustainability of global health, NCDs converge with infectious diseases and poverty in low- and middle-income countries (LMICs) to represent a particularly pernicious multidimensional burden of disease with significant implications for development (Bygbjerg 2012). This is only worsened as the behavioral risk factors for NCDs (alcohol, diet, physical activity, and tobacco) are inextricable from many of the aspirations and lifestyle shifts that accompany development. Although NCDs represent a powerful challenge to current models of global health governance, funding priorities, and paradigms, they also represent a springboard for potentially vibrant transdisciplinary concept building. This, in turn, could offer an important vehicle for contributions to critical studies of global health by geographers of all subdisciplines.

The recent Economic and Social Research Council (ESRC 2013) Benchmarking Review of UK Human Geography describes health geography as the “foremost” of the discipline's “nascent fields.” This vibrancy can be traced back to the 1990s and a concerted movement away from medical geography's concern with the distribution of disease, risk factors, and health care facilities to a closer connection with social and cultural geography's then-emergent interest in the body, the meaning of place, and social theory (R. Kearns 1993). This need to move beyond the “shadow of medicine” (R. Kearns and Gesler 1998, 3) and emerge as a reformed, critical health geography echoed the broader shift from a “biomedical model of disease” to a social model anchored in the “new public health” (Petersen and Lupton 1996; T. Brown and Duncan 2002; see also Parr 2004). It is interesting, therefore, to remember this cultural and theoretical turn in the subdiscipline, especially given that the conceptual concerns of many health geographers—choice, agency, and responsibility—remain real and ironic absences in the highly medicalized field of global health (Dry and Leach 2010; Clark 2014). In recent years, there have been a number of calls for greater geographical attention to global health (T. Brown 2011, 2014; T. Brown, Craddock, and Ingram 2012; T. Brown and Moon 2012; Herrick 2014; Herrick and Reubi 2016). This has been largely spurred by a drive to make collective sense of existing geographical writing on inherently related themes including (bio)securitization (Ingram 2005, 2009, 2013), the geopolitics of infectious disease (Craddock 2000; Harris Ali and Keil 2008), inequality and inequity (Pearce and Dorling 2009; G. Kearns and Reid-Henry 2009), livelihoods and the political ecologies of health (King 2010, 2011; King and Crews 2013), pharmaceuticals (Craddock 2012), geographies of care (Lawson 2007), globalization (Sparke 2009; Sparke and Anguelov 2012), the situated “making” of global health (Brada 2011), the spatialized logics of epidemiology (Laurie 2015), the commodification of bodies (B. Parry 2004, 2008), and immunity, infectious disease, and the nonhuman (Hinchliffe and Ward 2014; Hinchliffe 2015).

Moreover, given the current attention to NCDs, it is worth highlighting critical geographical engagements with their four major risk factors: obesity (Herrick 2007, 2009b; Evans, Crookes, and Coaffee 2012; Guthman 2012, 2013), alcohol (Kneale and French 2008; Jayne, Valentine, and Holloway 2010, 2011; Herrick 2012), smoking (Thompson, Pearce, and Barnett 2007, 2009), and physical activity (Herrick 2009a; Hitchings 2013; Latham 2015). Despite their clear relevance, however, only a handful of these papers explicitly reference global health debates. Global health is also a glaring absence in recent, well-received health geography compendia (T. Brown, Mclafferty, and Moon 2009; Gatrell and Elliot 2009; Anthamatten and Hazan 2011). At the most practical of levels, this means that this rich corpus of highly prescient geographical writing rarely appears in keyword database searches for global health literature, further reinforcing the discipline's absence. This is unfortunate, given the potential of so many geographers to both contribute to global health debates and raise the profile of the discipline within these. This research includes, but is in no way limited to, critical approaches to political economy, scientific knowledge, security and vulnerability, diplomacy and governance, changing paradigms of aid and development assistance, socioeconomic inequity, sustainability, embodiment, social justice, globalization, rights and responsibilities, risk and resilience, urbanization, socionature, and the dialectical relationships between people and places (see T. Brown, Craddock, and Ingram [2012] and T. Brown and Moon [2012] for excellent reviews). This is far from an exhaustive list but gives a sense that the potential for geographical contributions to global health's “existential challenges” (Garrett 2013) goes far beyond health geography alone. I now turn to one conceptual approach that offers the potential to make this contribution more explicit and, furthermore, might offer up a productive platform for better working across and between geography and anthropology.

## Geographical Contingency and Contingent Geographies

Global health's institutional location within medical and public health schools might have initially stymied its engagement with social theory, but this situation is rapidly changing. Kleinman's (2010) *Lancet* paper, for example, argued for the application of social theory to help students to “generalize knowledge and to develop a more systematic critical reflection on global health problems and programs as a complement to epidemiological, health services, policy and ethical studies” (1519). Leaving aside my concern with the words *generalize* and *systematic*, he drew particular attention to Merton's (1936) concept of the “unintended consequences of purposive social action,” as well as the anthropological framework of social suffering and structural violence (Farmer 1996a; Bourgois 2003). Together, these two concepts have proved exceptionally influential within critical studies of global health, especially for those anthropologists keen to highlight what happens when “the supposed beneficiaries of interventions are generally lost from view” (Biehl and Petryna 2014, 376). Here, however, I want to explore how the concept of contingency might be able to bridge current anthropological concern with the genesis and experience of suffering with the very real need to better understand how purposeful and well-intentioned actions can produce unintended and unanticipated consequences. In so doing, I wish to move beyond the idea of contingency as simply the workings or influence of local context so ably advanced in cultural anthropology and instead make a case for how geographic contingency might be read as the juncture of sociospatial conditions of possibility with uncertainty, chance, and fortuitousness. Moreover, these junctures often occur without design, such that they can produce new emergent and contingent geographies or the sociospatial formations whose existence is only made possible by the very contingency of their preconditions.

In many respects, exploring contingency necessitates a brief return to Kearns's oft-cited paper that highlighted the “dynamic relationship between health and place and the impacts of both health services and the health of population groups on the vitality of places” (R. Kearns 1993, 145). As he argued, exploring this dialectic requires “models of society as well as of health that recognize the contingent relations that pertain for individuals and groups at particular locations” (145). These ideas have further advanced efforts to theorize place in relational terms, where “individuals often influence, and are influenced by, conditions in multiple places” that, moreover, are produced and experienced through dynamic (and often distal) social and power relationships (Cummins, Diez Roux, and Macintyre 2007, 1828). Although Cummins and Kearns work from quite different epistemological perspectives, the shared geographical concern with the “processes and interactions” between people and places of varying degrees of proximity is important, as it helps introduce new “dimensions of incertitude” into global health research (Leach, Scoones, and Stirling 2010, 373). In this sense, then, I am not working with contingency in the sense of the measures that need to be put in place to mitigate and manage potentially negative effects of an intervention (i.e., a contingency plan). Instead, my geographical reading of contingency is one that starts from the belief that certain events, processes, or outcomes cannot be predicted or determined and their conditions of possibility are inextricable from the locales in which they arise (Barry 2014). Moreover, the complexities inherent within this uncertainty act as a marked challenge to a global health epistemology that demands logic and predictability over instability and emergence.

The uptake of complexity theory as an “explanatory schema” (Harrison, Massey, and Richards 2006) with great potentiality for the study of health (Curtis and Riva 2010) is worth briefly noting here for its concern with interactions, flows, networks, agency, relationality, nonlinearity, feedback, and emergence (Rickles, Hawe, and Shiell 2007). This helps challenge the polarized spatial logic of global health evinced in much anthropological writing—a global versus an embedded local—where social suffering emerges through the embodiment of distal processes outside individual control (Farmer et al. 2006). This eschewing of control reinforces a tendency among some anthropological writing to situate “whole communities within a discourse of victimization” (Panter-Brick 2014, 439). This focus tends to underplay the novel forms and processes of emergence charted by complexity theory that have become new, “unanticipated anthropological terrain” and that also characterize the “profound disconnections” between “campaign designs and intentions and the complex ways in which those campaigns are actually received and critiqued” (Biehl and Petryna 2014, 380). Although anthropologists have proved particularly adept at chronicling the experiences of this disconnect (Bourgois 2003; Nguyen and Peschard 2003; Scheper-Hughes 2004; Livingston 2012), they have tended to pay less attention to the relational and, indeed, the broader sociospatial genesis and consequences of disjuncture (however, see Crane 2013).

By contrast, here I both draw on and critique H. Brown and Kelly's (2014) recent work on “hotspots” of viral hemorrhagic fevers (VHFs) as producers and products of “radical and contingent relationality” (292) that “[defies] scalar logic” (283). Their emphasis on the “spatio-temporal heterogeneity” and “socio-political substance” (282) that characterizes the conditions of emergence for infectious disease is significant and helps us uncover what they termed “the elaborate temporal and material relationalities that cultivate networks of pathogenic exchange” (288). Although their work is a powerful contribution to our conceptual armory for exploring the outbreak of viral infections such as VHFs, it is of less use in the context of NCDs, which have arguably more politically and economically contentious pathogens (i.e., alcohol, food, tobacco) and processes of pathogenesis. This said, their assertion that outbreaks emerge amid “radical and contingent relationality” (292) offers an important starting point for exploring the role of geographic contingency in explaining why “universal” global health solutions—in this case the “best buy” of increased alcohol taxation—might produce not only unintended consequences but also emergent and unpredictable sociospatial formations that then act as a visceral critique of the very policies from which they have (directly and indirectly) emerged.

Contingencies can be thought of as instances that “interrupt the operation of processes, thereby producing different empirical outcomes in different contexts” (Jones and Hanham 1995, 186); in other words, new and unanticipated forms of emergence. The complicating factor here is that the “processes” of so many global health programs are merely either assumed or extrapolated from the limited global compendium of “evidence of best practice.” Contingency, therefore, might “signal the possibility of multiple outcomes derived from similar causal processes due to the complexity of social relations embedded in spatially differentiated contexts” (Jones and Hanham 1995, 186). When these “causal processes” themselves are the products of inherently imperfect epidemiological prediction that is often the product of a very narrow geographic imagination, however, the range of possible “differentiated outcomes” (186) becomes unfathomable. Moreover, this becomes even more complicated if we subscribe to the belief that “place contexts—with their differences in social, political, economic, and environmental characteristics—are obvious *locations* for the production of contingencies” (190). Geography, as ever, matters. This also means that Botswana is more than just the context of policy deployment; it matters deeply because of how, who, what, and where it is.

Contingency is consequently not only inherently geographical but, in so being, sits uneasily with a biomedically framed global health enterprise in which the “unexpected constitutes a threat … [and] carries with it an approach toward the unexpected which gives pride of place to the quantification of uncertainties” (Malaby 2002, 287). Places, then, can act both as conduits for and the emergent outcomes of contingency. This idea was recently explored by Amin (2013) in his work on the contingent vitality of cities of the Global South in which he made a claim for the strategic importance of the “specificities of location” (141) as a tool for the amelioration of some of the most pressing urban problems. Moreover, he argued, the fact of urban dwellers of the Global South being in the “practiced habit of living in particular circumstances of lack and uncertainty” (146) means that uncertainty is not erased or neutralized in and by everyday life but rather (and necessarily) worked with (see also Simone 2010) in the production of new, contingent geographies. Although I do not wish to glamorize the quotidian tedium and peril that can be produced by living with uncertainty, these ideas draw attention to the geographical foundations of contingency and hint at the productive nature of the emergent contingent geographies that they might inadvertently produce and to which I now turn.

## The Multiple Contingencies of Alcohol Taxation in Botswana

Alcohol occupies a liminal space within the global health landscape. On the one hand, its use, abuse, and health consequences are virtually absent from the programmatic priorities of the world's most significant global health philanthropies (Casswell and Thamarangsi 2009). On the other, mounting critique of this lacuna has invigorated the advocacy efforts of the global alcohol control movement, especially in the Global South. With alcohol now framed as “a global health problem” and a profound challenge to sustainable development (Beaglehole and Bonita 2009; WHO 2011), attention has turned to public health interventions to reduce population-level consumption, particularly in high-risk countries of the Global South. Central to the alcohol control community's lobbying efforts has been the promulgation of a simple message: evidence-based policy “best buys.” Interestingly, in its fear of the “problem deflation” tendencies of ethnographic research on alcohol (Room 1984), the alcohol control community has not only made its view on anthropology clear, but also eschewed contingency as inconvenient to the implementation of its universal public health policies (Room, Babor, and Rehm 2005; Herrick forthcoming). Its global “best buys” focus on the control and restriction of alcohol supply and advocate for limited opening hours and outlet density, increasing taxation, government monopolies on retail sales, increasing age limits, drink driving counter-measures and advertising/ marketing restrictions (World Health Organization 2010). In this paradigm, demand-side interventions such as education campaigns or labeling are rejected as unscientific and nonscalable (see, e.g., the debate in Craplet 2006; Rehm, Babor, and Room 2006) or as unwelcome evidence of industry influence (Casswell 2013).

With this context in mind, Botswana—a midsized, land-locked country whose significant mineral wealth accounts for roughly 40 percent of all government revenues—serves as a particularly interesting location for an exploration of alcohol and global health for two reasons. First, it occupies a paradigmatic place in anthropological writings on global health through Livingston's fascinating corpus of work documenting the profound sociobiological shifts that have occurred alongside the country's emergence as a rare postindependence African “success” story (Livingston 2005, 2008, 2012; see also Brada 2011). In particular, her work on cancer reminds us how chronic disease has for too long been viewed as “an esoteric distraction from more pressing concerns in global health,” such as HIV/AIDS (Livingston 2012, 9). Yet, and as she powerfully argued, increasing rates of cancer are actually a deeply contingent artefact of “African health *after* antiretrovirals” that highlight the extent to which biomedicine can only ever be an “incomplete solution” to complex global health problems (Livingston 2012, 7). Second, Botswana has arguably spearheaded the current southern African trend toward the stricter regulation of alcohol. This trend cannot be dissociated from work undertaken by the WHO in singling out the African region as lagging behind in the uptake of alcohol taxation (WHO Regional Office for Africa 2008). Their recommendations for taxation have been justified through numerous public health publications highlighting the urgency of the region's alcohol scourge and the relative inadequacy of the current policy response (C. Parry 2014; Ferreira-Borges et al. 2015; Jernigan and Babor 2015; C. Parry et al. 2015) in the context of the predatory tactics being used by the global liquor industry to gain market share on the continent.

Alcohol has long played a role in southern African life. Chibuku, a traditional, low-alcohol sorghum beer, has, for example, been consumed in various guises across the region for at least the last century (van Wolputte and Fumanti 2011). More recently, however, alcohol has been implicated in the widespread moral panic over the spread of social and economic “ills,” including violence, crime, injury, unemployment, and HIV/AIDS (Obot 2006). When teetotal President Ian Khama took office in 2008, he acted on his “personal distaste for alcohol,” concern with national “moral weakness,” and lack of discipline (Burgis 2009) by unilaterally imposing a 30 percent levy on all alcohol and warning that this would ultimately be increased to 70 percent (Good 2010b, 321; Gulbrandsen 2012).[Fn en0002] According to Khama, “[Alcohol] is an *enemy*, anything that causes people to lose their lives unnecessarily before their time must be an enemy” (Burgis 2009, italics added). Despite being challenged in court by the country's largest brewer, the levy was quickly increased to 40 percent in 2010, 45 percent in 2012, and 55 percent in 2014 after a government-commissioned report revealed the resilience of public consumption (Pitso and Obot 2011). The levy has been combined with significant fines for drunk driving (roughly 1,000 Pula or $90), promises of more active policing, strict controls on drinking in public places, restrictions on bar opening times, the regularization of traditional beer production and retailing from “depots,” and an (informal) ban on local television advertising. Botswana's comprehensive uptake of so many of alcohol control's “best buys” thus makes it an exceptionally important global health test bed. Moreover, and as I explore, it also exemplifies how geographical contingency and the genesis of new and emergent contingent geographies fundamentally call the public health faith in universally applicable public health interventions into question.

Health economists argue that alcohol, like tobacco, is a commodity for which demand is fairly price elastic (Elder et al. 2010). Thus, it is assumed that as prices rise and affordability declines, population-level rates of consumption and, therefore, alcohol-related harms will also fall (Wagenaar, Salois, and Komor 2009; Wagenaar, Tobler, and Komro 2010). Although this advice is presented as universally applicable, it is important to remember that the evidence called to the service of such arguments is often drawn solely from an exceptionally limited number of Global North case studies (Herrick forthcoming).[Fn en0003] Just as with other (global) health interventions, the aspatialized and simplified evidence used to justify alcohol taxation is itself, ironically, a product of multiple geographical contingencies. Moreover, the diffuse ways in which taxation is understood and acted on by the public reveals the extent to which “people are plural beings and not reducible to ‘populations,’ and local realities still very much frame, constrain, and orient interventions” (Biehl and Petryna 2014, 385). Yet, although local realities are undoubtedly important, the alcohol levy represents more complex sets of confluences that tend to “defy scalar logics” (H. Brown and Kelly 2014, 283) in efforts to manage the “contingent convergence of pathogenic potential” (292). Indeed, instead of managing the risks and uncertainties engendered by alcohol consumption, the convergence of the levy with sociospatial processes unique to Botswana offers up the conditions of possibility for emergent geographical formations. The point is that both geographical contingency and the contingent geographies that result cannot be predicted by the methodological arsenal of epidemiology; they are instead the outcome of the happenstance that is coproduced in unique and opportunistic ways by the dialectic between people and places.

To explore these ideas in more depth, in 2014 I undertook twenty interviews with alcohol (policy and industry) stakeholders in Gaborone and conducted 300 surveys across five of the city's districts exploring the levy's effects on attitudes toward drinking and consumption behaviors. The survey research was undertaken with the University of Botswana with the aim of collating basic demographic data and exploring individual motivations for drinking, consumption habits, how respondents' drinking practices have changed through time, attitudes toward the levy, the effect of the levy on respondents' drinking practices, and respondents' perceptions of its effects on other's practices, as well as perceptions of the nature and extent of Botswana's “alcohol problem.” The survey was piloted, collectively refined, and readministered by three research assistants in either Setswana or English, depending on the preference of the respondents. The same survey was administered across the city and the research assistants recruited respondents from a variety of drinking establishments across middle- and low-income neighborhoods to talk to customers. This purposeful sample was chosen to ensure the recruitment of drinkers, rather than nondrinkers, to better gauge the influence of the levy on attitudes toward drinking and consumption habits (something that nondrinkers would be less able to answer). The survey was administered over the course of two weeks and responses were recorded by hand in the field for reasons of security and convenience.

The interviews were undertaken with a variety of policy and industry actors, with respondents recruited through contact networks and snowballing techniques. As might be imagined in a capital city of only 200,000, the worlds of alcohol production, distribution, retail, and regulation are fairly small, so the sample size represents a very thorough cross section of key actors. The semistructured interviews each lasted around an hour and were audio recorded (where consent was given), usually in respondents' places of work. The interviews aimed to elicit a sense of the multiple dimensions of the alcohol control debate, the arguments for and against the levy, and perceptions of its consequences. The interviews thus act as a counterpoint to the public and lay opinions evinced in the survey findings, exploring the rationales for the implementation of the levy from governance and public health perspectives and the challenges that it poses to the private sector. Given the autocratic nature of government in Botswana, it was unsurprising that respondents requested anonymity and many were reticent to directly criticize the president, despite discussing a policy tool that was very clearly his personal moral pursuit. This fear of directly criticizing the leadership was compounded by the small social circuit that respondents tended to move within. In the text, anonymity is maintained and context deepened by identifying respondents by their domain of work.

**Figure 1.**
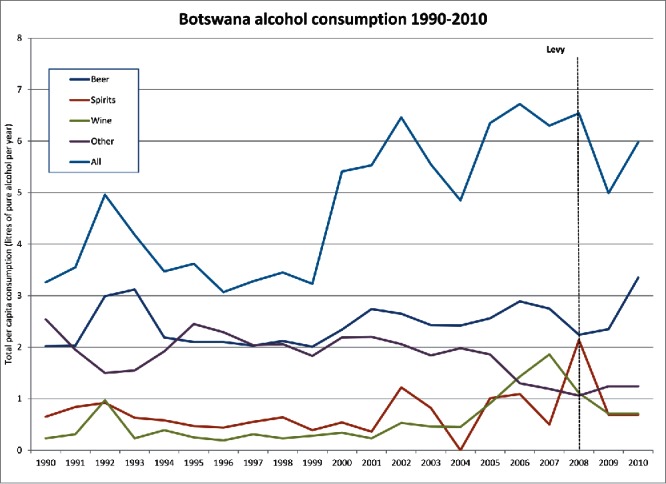
Alcohol consumption in Botswana, 1990–2010, in liters per person per year. *Source*: World Health Organization Global Information System on Alcohol and Health (2015).

Here I want to explore the effects of the levy through the lens of geographical contingency before examining their emergent contingent geographies. Somewhat unhelpfully given the broader demonstration potential inherent in such a large-scale public health intervention, no baseline data were collected on drinking rates by the Ministry of Health before the levy's imposition. Press reports in Botswana (Gaotlhobogwe 2013) were, however, quick to pick up on WHO data showing that drinking rates were already on a downward trend between 2000 and 2005 when the levy was first mooted as part of President's Commission of Inquiry into the “social vices” gripping the country. The WHO Global Information System on Alcohol and Health (GISAH) does show that consumption levels dipped the year after the 2008 imposition of the levy (see Figure 1) from 6.54 to 4.99 liters per person per year. By 2010, the last year for which WHO data are available, drinking rates had again risen by almost 20 percent to 5.98 liters per capita. This rebound was corroborated by industry stakeholders who noted a substantial drop in volume sales as the initial price shock of the levy took effect. As one interviewee thus noted,

With the introduction of the levy it was quite a shock because the product got very expensive very quickly, initially we saw a decline in volumes, but I don't think that is the case anymore. (Alcohol producer, interview, 2014)

Since 2009 and despite annual rises in the levy rate, however, not only has per capita consumption been steadily climbing according to industry sources, but drinking habits themselves have changed. As another industry interviewee asserted,

As the levy has gone on and increased, people have carried on moving downwards [in value terms] in what they buy, so my business hasn't declined at all, it's just shifted. (Alcohol distributor, interview, 2014)

As prices have gone up, value sales might have suffered, but volumes of cheaper drinks have remained relatively steady. This could be due to a reported surge in home drinking where “they [now] go to bottle stores as it's cheaper” (Public health policymaker, interview, 2014). Here, they can now pick up “DIY Chibuku” packs for home consumption in a move designed by one major national brewer to offset the effects of traditional beer depot closures and concomitant falls in their Chibuku sales. In asking about the consumer response to the constant upward trend in alcohol prices, respondents from the liquor industry highlighted a turn from market-leading beer St. Louis (3.5 percent alcohol by volume [ABV]) to higher strength Carling Black Label (5 percent ABV), with Black label “Sharepacks” (750 ml bottles rather than the 340 ml more traditionally purchased) now enjoying the greatest sales growth. These formats are seen as representing better alcohol unit-per-Pula value but also represent new sources of risk as, unlike other countries in the region where large bottles are shared among drinkers, Batswana tend to consume their own. Such trends were also leading to a “neglect of the home in favor of drinking” (Alcohol distributor, interview, 2014), with repercussions for the same domestic violence and poor economic productivity that the levy was brought in to combat.

The levy has also precipitated a collapse in the capital's formal nightlife due to restrictive opening hours and prohibitive pricing, much to the dismay of the city's musicians and young people. Some industry interviewees were also (unsurprisingly) swift to offer anecdotal evidence of a rise in drug use, a flood of cheap nonbranded alcohol imports from India, and the development of a significant cross-border liquor trade (alcohol distributor and retailers, interviews, 2014). With prices in South Africa over 60 percent cheaper than in Botswana and the Mafoeking branch of wholesaler Makro only a couple of hours' drive over the border, professional bootleggers and couriers have begun taking advantage of relatively lax border controls (trade association, interview, 2014). As one NGO interviewee noted:

If you ask government, they'll say that the money from the levy is going down so people must be drinking less, but there are all these villages along the border where people are just going across and buying alcohol. If you go over the border on a public holiday, the queues of cars coming back over the border, filled with booze …

Another from an industry trade association did little to hide his complicity in this trade:

I buy from South Africa by courier; you only need to pay 500 Pula. I buy a box of champagne, in South Africa is only 2,000 Pula, in Botswana it is 3,500 Pula.[Fn en0004]

Industry representatives also noted a trend toward underinvoicing imported liquor brands at customs, for truckers to sell cheap South African–bought liquor to highway bottle stores, and for shipping containers of imported alcohol to go “missing” en route to Botswana, something industry sources termed *round tripping*. The alcohol levy is a thus a good example of the contingent emergence of significant, new, and unanticipated forms of human agency, commercial opportunity, and risk taking that has not yet been explored by the public health community even while they continue to argue for the universal efficacy of taxation (Elder et al. 2010).

These effects of alcohol taxation could not have been foreseen by the “evidence” used in the formulation of evidence-based policy. Instead, they have emerged at the junctures of sociospatial conditions of possibility with uncertainty, chance, and fortuitousness of which I highlight two clear examples. First, Botswana's location on the South African border means that it shares its powerful neighbor's liquor distribution networks and South African manufacturers enjoy a significant market presence. Moreover, the value differential between the Rand and the Pula has precipitated bootlegging allied to a cross-border drug trade that has, in turn, reportedly led to new forms of substance abuse: “a rise in self-mixtures, cough mixtures (codeine) and coke, sleeping tablets” (addiction services NGO, interview, 2014). Furthermore, the proximity and cultural influence of Johannesburg has reinforced disquiet with the ways in which pricing structures deny aspirant Gaborone residents access to the brands and lifestyles enjoyed by South Africans. This influence has, in turn, reinforced the resilience of consumption for, as one industry interviewee remarked, “Humans are humans and they'll make a plan.” Second, there are also interlocked geographical contingencies inherent in President Khama's military education at Sandhurst after the interracial marriage of his parents forced them to flee Southern Africa and live in exile in the United Kingdom. The same British education might have precipitated his fetishization of “puritanical discipline” (reports abound of his daily 4:30 a.m. workouts) that he later brought back to Botswana. Finally, there is his faith and inherent dislike of alcohol (there is much speculation about a history of alcoholism in his family), a stance that finds many willing ears in a country where rates of lifetime abstention are high and 70 percent of people self-identify as Christian. This particular amalgamation of geographical contingencies has produced such a moralized policy environment that “the message coming out from government is stay sober and this especially alienates young people who don't want necessarily to stop drinking” (addiction services NGO, interview, 2014).

The multiple disconnects between the presidential discourse of alcohol as “social scourge,” a biomedical framing of alcohol as risk, and the ambiguity of public opinion is borne out in the survey findings. For example, despite the media attention on the levy and related messaging around the evils of alcohol, only just over half of the survey respondents (55 percent) believed alcohol to be a “problem” for the country. This chimes with the 20 percent of respondents who claimed to be drinking more now than ever before. Yet, in contrast to WHO data suggesting a rebound in consumption, 50 percent of survey respondents still stated that their alcohol consumption had declined in the last five years. This is then notable because 60 percent of respondents believed that other people had not decreased their alcohol intake since 2008, highlighting the extent to which gathering data on alcohol consumption is fraught with bias and self-reporting inaccuracies (Boniface and Shelton 2013). Further dashing the hopes of health economists, even though 71 percent of respondents thought the levy already too high, 78 percent of these stated that they would continue to drink even if it was further increased. This compulsion was particularly strong among those respondents who stated that they now drank as an act of political resistance against an autocratic government that has overseen mounting rates of youth unemployment and visible inequality (Good 2010a). This sentiment was also picked up by the opposition party leader in the run-up to the 2014 elections in his assertions that “Batswana essentially consume liquor because of the contempt they have for Khama … [he] has taken away taste and mystique of the forbidden fruit and now Batswana want back the taste” (Bagwasi 2015).

It must not be forgotten that alcohol's introduction has also cross-cut profound shifts in urban lifestyles, as new malls emerge across the Gaborone skyline, a new central business district rises, and the cost of living soars. The disparity between the capital and rural life is becoming ever more marked, bringing new and unpredictable societal concerns. Importantly, and as so often ignored in the biomedical framing of alcohol, these shifts go beyond public health to incorporate questions of livelihoods, agency, coping, and resilience (Herrick 2013). Moreover, they have also carved out new contingent geographies, or spaces where the effects of the levy coalesce and are amplified into new topological formations across Gaborone's rapidly changing urban landscape. These include the opportunistic and rebellious spaces created by illegal after-hours drinking and “car pimping” (partying with music blaring from parked cars) now found in the city's numerous scrubby open spaces under cover of darkness and organized through social media (Pritchard 2015). There has been a rise in music and drinking festivals located far into Gaborone's rural periphery and thus removed from the gaze of urban enforcement agencies. These human responses to the restrictions imposed by the levy are not only made possible by Gaborone's unique topography but are accompanied by unanticipated forms of risk. For example, the levy has created spaces of necessity and innovation for new spatial networks of cross-border trade, smuggling (both informal and commercial), and new liquor distribution routes designed to try and minimize the commercial damage of the levy to industry. Finally, the levy has brought emergent spaces of policy and knowledge creation into being that reference international experts and case studies of best practice in the Global North, sideline regional knowledge (and especially, as interviews revealed, the South African experience), and discount the uncertain outcomes that characterize contingency as epidemiological inconvenience (see Crane 2013).

These new spatiotemporal topologies represent neither the successful deployment of the levy, nor are they merely symptoms of its failure. Rather, and instead, such contingent geographies represent the resilient adaptation of pleasure-seeking behaviors through the opportunities afforded by conditions of multiple uncertainty. This resilience is inadvertently underlined by Khama's (2015) State of the Nation Address, which noted that the levy had produced revenues of P1,867,586,562 since 2008 and that “a 2012 evaluation of the impact of our alcohol reduction campaign interventions indicated that there was a reduction in alcohol consumption from 8 litres per capita to 7 litres.” At the time of interviewing, no respondent could explain how the funds had been disbursed and many suspected that they were being used to prop up general government revenue rather than being channeled into the alcohol harm-reduction programs promised as part of the levy's rationale. It is notable, therefore, that the ever-mounting levy revenue, its annual increase, and data suggesting that consumption has actually climbed since 2010 to 7 liters per capita coalesce to confirm that contingency should never be dismissed as either epidemiological inconvenience or simply the workings of local context. The obvious policy irony is that the levy funds represent a very significant income stream, the maintenance of which relies on the persistence of drinking and the success of which is generally couched in terms of the fund's size rather than evidence of any positive social changes brought about by the levy. Alcohol taxation is promoted in ageographical terms, yet as these findings have shown, the hope of universality can only ever be a geographical fallacy. The contingent geographies of Batswana drinking highlight the depth and extent of human agency and tenacity as well as how complexity perpetually destabilizes biomedical faith in global health “solutions.”

## Conclusion

Global health programs are largely and necessarily predicated on a great faith in the power of quantification, prediction, and evidence. Global health is experienced and enacted in grossly unpredictable ways, however, and therefore actively produced in a complex set of interwoven engagements and interrelationships. Tracing these emergent processes is essential to understanding where global health might fail and also where it might succeed. As the uptake of global health as an object of study and a type of praxis grows within universities, geographers have the conceptual tools to be central to this. As I have argued, though, geography has been sidestepped despite the call for the integration of global health with the “socializing disciplines” (Farmer et al. 2013). Although geography falls within this label, the potential it offers to the changing agendas of a reformulated post-2015 landscape of global health remains underrealized. As I have argued, this could in part be traced to health geographers' highly successful creation of a subdisciplinary niche and the resultant siloing of health research within this. Thus, if this article represents a call of any kind, it would be for a greater degree of reflexivity by geographers of all subdisciplinary persuasions on the (post-2015) global health significance of their research. This means going beyond the biomedical confines of diagnosis, disease, and treatment to think about health in metonymical terms: as representing both cause and consequence of multiple, entwined social, economic, security, environmental, political, and cultural issues. This is arguably even more significant in the complex world of global health, especially given that the “politics of contingency” have been so silenced in the quest for biomedical certainty (Malaby 2002, 307).

The production of new, contingent geographies by global health programs and rationales is consequently an area that merits distinct exploration. Using the example of Botswana, I have aimed to briefly sketch one such instance where interventions have reshaped the broader geographies not only of dwelling but also of doing business, evading regulatory efforts, and justifying political rationalities. This is not simply about locating global health with far “greater specificity” in intellectual and political terms than it has been to date (Holmes, Greene, and Stonington 2014, 476) but rather an argument for theorizing global health interventions as creative forces that rarely delimit uncertainty but instead produce emergent sociospatial properties (Gatrell 2005). The “changing landscape created by global health initiatives” (Biehl and Petryna 2014, 386) could therefore sometimes represent biomedical failure (Clark 2014), but it is also socially and spatially generative in ways that speak clearly to the recent work of Simone (2010) and Amin (2013) on the ability of people and places to resist, evade, and exceed governance efforts. Indeed, exploring contingency foregrounds how and why the global health enterprise generates the kind of “hybrid novelties, amplified reverberations, unanticipated lurches, and unintentional developments that escape intentional governance” explored in Botswana (Amin 2013, 151). Yet, as Pigg (2013) noted, “the questions arising from a hyper-consciousness of contingency and practice across scales and contexts of action can appear to those in the global-health mainstream as (variously) unproductive, unnecessary, overly abstract, too ambiguous, or off-topic and therefore not really useful for moving forward with getting things done” (133). In seeking to mitigate this persistent tension between interrogating and overcoming uncertainty, geographical perspectives on global health must harness contingency as a conceptual tool not simply for further critique but for getting important things done.

## Notes

1. It is interesting to note the following numbers of Academia.edu theme subscribers as a rough guide to subdisciplinary significance (figures as of 1 July 2015): human geography (26,297), cultural geography (10,898), medical geography (539), health geography (522), medical sociology (5,145), medical anthropology (20,650), global health (7,269), and public health (135,027).

2. In addition to excise duty, which cannot be changed by the individual member states of the Southern Africa Development Community.

3. Wagenaar, Tobler, and Komro's (2010) meta-analysis, for example, cites fifty studies, all of which come from either the United States, Canada, Finland, or Switzerland, all remarkably different contexts to the African countries for whom alcohol taxation is being pushed.

4. At the time of research £1 = Pula 10, $1 = Pula 6.25.
